# Obstetrical risk factors of ELBW

**DOI:** 10.1186/1824-7288-41-S1-A35

**Published:** 2015-09-24

**Authors:** Nicola Rizzo, Giuliana Simonazzi, Alessandra Curti

**Affiliations:** 1 Obstetrics and Prenatal Medicine – S.Orsola Hospital, Alma Mater Studiorum – Bologna University, Italy

## 

Preterm birth is the leading cause of perinatal morbidity and mortality worldwide [1]. It contributes to 70% of neonatal mortality and approximately half of long-term neurodevelopmental disabilities [2]. The obstetrics precursors leading preterm birth are delivery for maternal or fetal indications, spontaneous preterm labour with intact membranes and preterm premature rupture of membranes (pPROM) [3]. It is estimated that about 30-35% of all preterm births are indicated, 40-45% follow spontaneous preterm labour and 25-30% occur after pPROM [3].

Premature infants born at a gestational age of 32 weeks or less are obviously at greatest risk. The term “*extreme low birthweight*” (ELBW) is used to identify newborns with birthweight less than 1000 g. Although their prevalence is less than 1%, these newborns disproportionately account for nearly one-half of all perinatal deaths [4]. 

Antecedent risk factors for ELBW neonates, though geographically heterogeneous, include nulliparity and multiple gestations, each accounting for one-third and one-fourth of all births, respectively [5]. Spontaneous preterm labor precedes 34% of these deliveries and premature rupture of membranes in 25%. The pregnancy is complicated by hypertensive disease in about 20% of cases and bleeding and chorioamnionitis in 18%, respectively. Moreover, small for gestational age infants rate ranged from 16 to 20%. When the frequencies of these factors is compared between the United States and other countries, PROM rate is similar between the groups (25% vs. 26%, respectively), while others are not (chorioamnionitis: 18% vs. 37%, respectively). These variations may be due to publication bias, differences in maternal demographic characteristics, differences underlying burden of maternal or fetal illness, and/or differences in obstetrical practice patterns.
